# Defunctioning stoma and short- and long-term outcomes after low anterior resection for rectal cancer—a nationwide register–based cohort study

**DOI:** 10.1007/s00384-021-03877-y

**Published:** 2021-03-17

**Authors:** Soran Gadan, Judith S. Brand, Martin Rutegård, Peter Matthiessen

**Affiliations:** 1 Department of Surgery, Örebro University Hospital, Örebro, Sweden; 2 Department of Surgery, Faculty of Medicine and Health, Örebro University, Örebro, Sweden; 3 Clinical Epidemiology and Biostatistics, School of Medical Sciences, Örebro University, Örebro, Sweden; 4 Surgical and Perioperative Sciences, Surgery, Umeå University, Umeå, Sweden; 5 Wallenberg Center for Molecular Medicine, Umeå University, Umeå, Sweden

**Keywords:** Anastomotic leakage, Defunctioning stoma, Rectal cancer

## Abstract

**Purpose:**

A defunctioning stoma reduces the risk of symptomatic anastomotic leakage after low anterior resection for rectal cancer and mitigates the consequences when a leakage occurs, but the impact on mortality and oncological outcomes is unclear. The aim was to investigate the associations of a defunctioning stoma with short- and long-term outcomes in patients undergoing low anterior resection for rectal cancer.

**Methods:**

Data from all patients who underwent curative low anterior resection for rectal cancer between 1995 and 2010 were obtained from the Swedish Colorectal Cancer Register. A total of 4130 patients, including 2563 with and 1567 without a defunctioning stoma, were studied. Flexible parametric models were used to estimate hazard ratios for all-cause mortality, 5-year local recurrence, and distant metastatic disease in relation to the use of defunctioning stoma, adjusting for confounding factors and accounting for potential time-dependent effects.

**Results:**

During a median follow-up of 8.3 years, a total of 2169 patients died. In multivariable analysis, a relative reduction in mortality was observed up to 6 months after surgery (hazard ratio = 0.82: 95% CI 0.67–0.99), but not thereafter. After 5 years of follow-up, 4.2% (173/4130) of the patients had a local recurrence registered and 17.9% (741/4130) had developed distant metastatic disease, without difference between patients with and without defunctioning stoma.

**Conclusion:**

A defunctioning stoma is associated with a short-term reduction in all-cause mortality in patients undergoing low anterior resection for rectal cancer without any difference in long-term mortality and oncological outcomes, and should be considered as standard of care.

## Introduction

In the past decades, the survival rate after rectal cancer treatment has increased and local recurrence rates have decreased. Important reasons for these achievements have been the introduction of total mesorectal excision (TME) and neoadjuvant therapy [1–3]. In patients with low and mid rectal cancer, TME with an anastomosis at the pelvic floor, also labeled as low anterior resection of the rectum (LAR), is currently the standard surgical procedure. Despite these treatment advancements, adverse events are common with the most feared being anastomotic leakage, which often has been reported to occur in approximately 10 to 12% of all patients undergoing LAR [4–6]. Possible consequences of anastomotic leakage include pelvic sepsis, the need for urgent reoperation, multi-organ failure, increased short-term postoperative mortality, and an increased risk for a permanent stoma [7, 8].

The main reason for constructing a defunctioning stoma in LAR is to decrease the risk of symptomatic anastomotic leakage, as well as moderating the adverse consequences when anastomotic leakage occurs despite defunctioning of the low colorectal anastomosis [9–11]. Several studies have demonstrated the benefit of a defunctioning stoma, as reflected by lower rates of symptomatic anastomotic leakage, urgent reoperation, and early postoperative mortality [9, 10, 12–14]. For these reasons, the use of routine defunctioning stoma in LAR has become widespread practice. Nevertheless, a defunctioning stoma is also associated with various adverse events including high stoma output, dehydration, bowel obstruction, parastomal hernia, and an increased risk of stoma permanence [15–21]. To date, few studies have examined whether or not a defunctioning stoma influences long-term oncological outcomes. Current evidence regarding the potential long-term impact of symptomatic anastomotic leakage in patients undergoing LAR is also conflicting [22–25].

The aim of this large population-based study using nationwide register–based data was to compare the short- and long-term outcomes in patients undergoing LAR with and without a defunctioning stoma in terms of all-cause mortality, local recurrence, and distant metastatic disease.

## Methods

The present study uses data from a national register–based cohort of patients who underwent anterior resection for rectal cancer in Sweden between 1995 and 2010, all registered in the Swedish Colorectal Cancer Registry (SCRCR). The SCRCR is a national quality register that prospectively records information on variables related to patient demography, surgical and perioperative details, neoadjuvant and adjuvant treatment, postoperative events, and short- and long-term oncological outcomes. Nearly all patients (99%) diagnosed with adenocarcinoma of the rectum in Sweden have been registered since the start of the register in 1995 [26]. Some validation studies of the SCRCR have been conducted with the latest report, Moberger et al. in 2018, demonstrating high accuracy for most relevant variables [26–28].

In this study, the aim was to include exclusively patients having undergone sphincter-saving TME surgery with an anastomosis on the pelvic floor, also labeled as LAR. Between 1995 and 2010, a total of 19,359 patients underwent abdominal resection for rectal cancer in Sweden: anterior resection (*n* = 10,811), abdominoperineal excision (APE; *n* = 5938), and Hartmann’s procedure (*n* = 2610). Only patients undergoing anterior resection were assessed for the present study. Anastomotic height is not a registered variable in the SCRCR and therefore, tumor height was deemed the best proxy for anastomotic level.

**Fig. 1:**
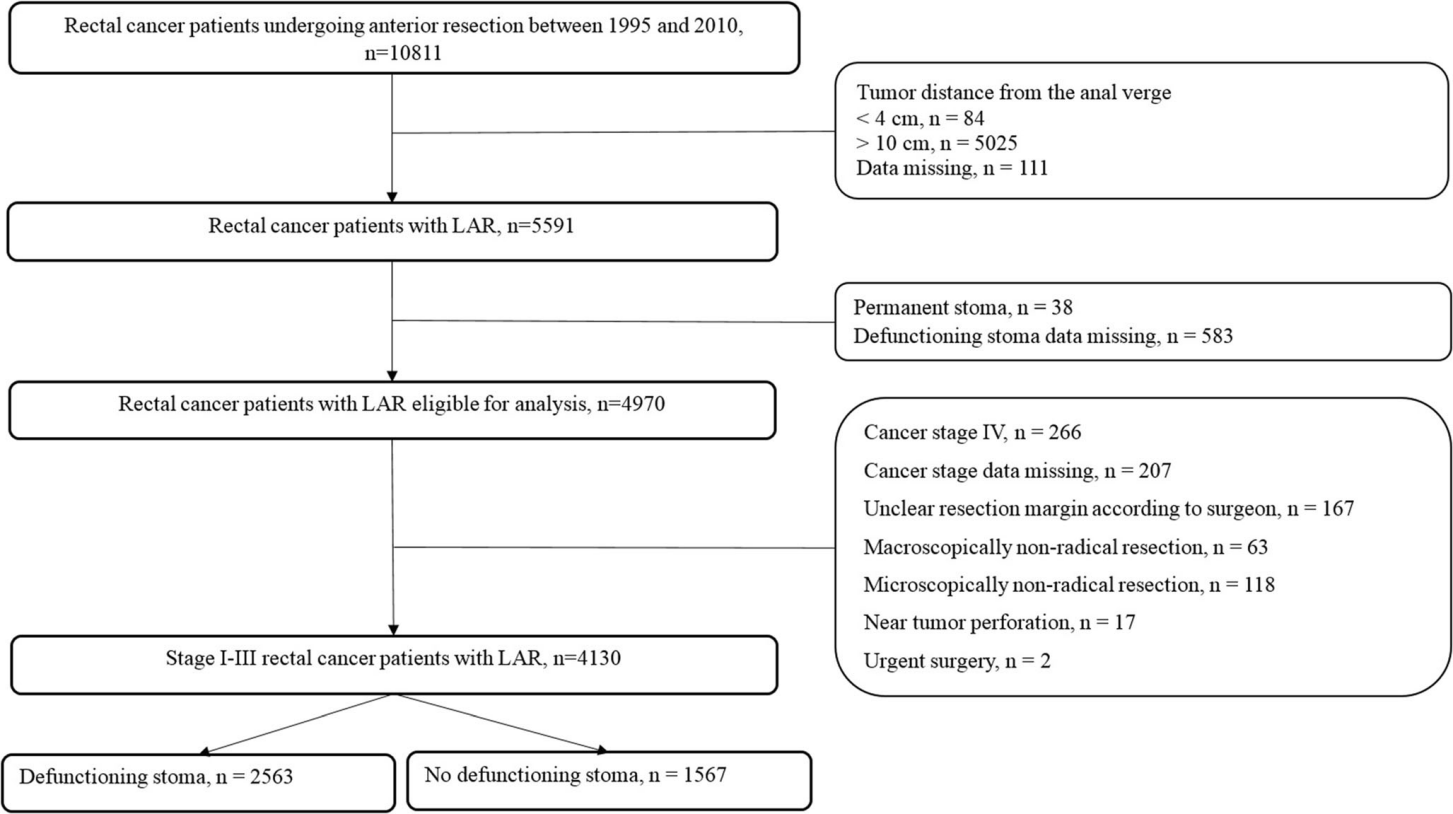
Flow chart of the study population of patients operated with low anterior resection for rectal cancer in Sweden between 1995 and 2010. Defunctioning stoma was registered in the SCRCR by all health care regions from 1998 onwards

Patients with a tumor level of 4 to 10 cm from the anal verge were identified and included. Patients with a tumor level of ≥ 11 cm (*n* = 5025) were deemed less likely to have an anastomosis at the pelvic floor, and were therefore excluded. Patients with a tumor level of ≤ 3 cm above the anal verge were arbitrarily excluded (*n* = 84) as it was deemed that there was a potential risk of misclassification (APE, or possibly Hartmann’s procedure, incorrectly registered as LAR). We further employed the following exclusion criteria: cancer stage IV, patients registered with macroscopically or microscopically not radical resection, unclear resection margin as assessed intraoperatively by the surgeon, bowel perforation near the tumor, emergency surgery, and cases where data on defunctioning stoma or tumor level were missing (Fig. 1). After these exclusions, the study population for analysis consisted of 4130 stage I–III rectal cancer patients having undergone curative LAR, including 2563 with a defunctioning stoma at index surgery and 1567 without defunctioning stoma at index surgery. Permission for the study was obtained by the Uppsala Regional Ethics Review Board, Uppsala, Sweden (Dnr 2014/470).

### Patient characteristics, operative details, and postoperative outcome

The following variables were extracted from the SCRCR for descriptive statistics and/or inclusion in multivariable analyses: age, sex, clinical cancer stage (cTNM), tumor level, neoadjuvant therapy, adjuvant therapy, and year of surgery. Perioperative variables included duration of surgery, intraoperative blood loss, defunctioning stoma at index surgery, postoperative adverse events including symptomatic anastomotic leakage, hospital stay, and postoperative mortality.

Surgical and medical complications diagnosed within 30 days of index surgery are registered in the SCRCR. The SCRCR has no formal definition of anastomotic leakage, and any leakage diagnosed within 30 days of index surgery should be registered. It can therefore be assumed that registered leaks were symptomatic/clinical, and that asymptomatic/subclinical leaks may have been registered only to a negligible degree.

From 2007 onwards, the SCRCR also records BMI and the American Society of Anesthesiologists (ASA) comorbidity grade. As our study population included patients operated between 1995 and 2010, this information was only available in 23% (951/4130) of the patients.

### Long-term outcomes

Information on all-cause mortality is available for all registered patients through linkage to the Swedish Cause of Death register, and 5-year data on local recurrence and distant metastatic disease are directly recorded in the SCRCR. Local recurrence is defined in the SCRCR as any recurrence of previously operated rectal adenocarcinoma located in the pelvic cavity [29]. In a validation report by Jörgren et al., including patients operated between 1995 and 1997, 89% of the local recurrence cases found in all medical records were also registered in the SCRCR [27]. To our knowledge, no published validation data exist for distant metastatic disease.

### Statistical analysis

Distributions of patient characteristics in relation to the presence of a defunctioning stoma or not were compared using chi-square tests for categorical variables, while Student’s *t* test and Mann-Whitney *U* test were employed for continuous variables, as appropriate. Study endpoints for analyses were time to death from any cause, time to local recurrence ,and time to distant metastatic disease. For each of these endpoints, follow-up time was defined from the date of index surgery (LAR) until the event, emigration, death, or date of last follow-up (31 December 2016), whichever came first. When analyzing time to local recurrence and metastatic disease, death was treated as a competing event and follow-up was additionally censored 5 years after the index surgery. Five-year cumulative incidence curves for local recurrence and distant metastatic disease to summarize absolute risks of disease recurrence in relation to defunctioning stoma are also presented.

Flexible parametric models were used to estimate the association between the use of a defunctioning stoma and each of the study endpoints [30, 31]. The proportional hazards assumption was tested using likelihood ratio tests of time by covariate interactions. As with the Cox regression model, flexible parametric models yield hazard ratios (HRs) as relative measures of association, but also allow the association with the exposure (i.e., defunctioning stoma) to vary over time (i.e., non-proportional). Under the proportional hazards assumption, HRs estimated with these models are similar to those obtained from the Cox model. In all models, time since surgery was the underlying time scale and a restricted cubic spline with five degrees of freedom was used for the baseline hazard. In case of non-proportionality, time-dependent effects were modeled by adding an interaction term with time using a second spline with three degrees of freedom. To facilitate comparison across analysis models, HRs were also reported at different time points following surgery, for each month during the first year, and at 2, 5, 10, and 15 years after surgery.

All analyses were adjusted for calendar year of surgery to account for cohort effects in defunctioning stoma prevalence as well as survival and recurrence rates during the study period (model 1). Analyses were further adjusted for all observed potential confounders in a multivariable adjusted model including age, sex, cancer stage, tumor level, neoadjuvant therapy, and year of surgery (model 2). A sensitivity analysis was also performed to evaluate possible residual confounding by BMI and ASA comorbidity grade, by repeating the multivariable adjusted model (model 2) in the subcohort of patients operated between 2007 and 2010 with additional adjustment for these variables (model 3). All data were analyzed using Stata version 16 (StataCorp, College Station, TX, USA).

## Results

### Patient characteristics, operative details, and postoperative outcomes

Patients with a defunctioning stoma at index surgery were younger (mean age 66.4 compared with 68.3 years), more often male (61.1% and 49.6%), had a lower tumor level (mean 8.1 compared with 8.5 cm), and more often received neoadjuvant therapy (76.8% and 62.9%), respectively, as compared with those without defunctioning stoma. The use of a defunctioning stoma at index surgery increased over time during the study period. Distributions of other patient characteristics were comparable between the two groups.

Patients with a defunctioning stoma had increased intraoperative blood loss (median 600 mL compared with 500 mL). The rate of symptomatic anastomotic leakage was lower in patients with defunctioning stoma compared to that in those without (9.5% and 14.7%, respectively). Hospital stay was longer for those with a defunctioning stoma at index surgery. Early postoperative mortality was lower in those with defunctioning stoma at index surgery as compared with those without, 30-day mortality was 0.9% and 1.8%, respectively, and 90-day mortality was 1.8% and 3.6%, respectively (Table 1).

**Table 1:** Patient characteristics, operative details, and postoperative outcomes in patients operated with low anterior resection for rectal cancer with and without a planned defunctioning stoma

	All	Defunctioning stoma		
	(*n* = 4130)	No (*n* = 1567)	Yes (*n* = 2563)	*p* value
Age (years), % (*N*)				
< 65	37.1 (1534)	34.3 (538)	38.9 (996)	< 0.001
65–75	39.4 (1628)	36.8 (576)	41.0 (1052)	
> 75	23.4 (968)	28.9 (453)	20.1 (515)	
Mean, SD	67.1 (10.6)	68.3 (10.8)	66.4 (10.5)	< 0.001
Male sex, % (*N*)	56.8 (2345)	49.6 (778)	61.1 (1567)	< 0.001
ASA comorbidity grade, % (*N*)*				
1	29.2 (302)	22.1 (23)	30.0 (279)	0.22
2	58.2 (602)	65.4 (68)	57.4 (534)	
≥ 3	12.6 (130)	12.5 (13)	12.6 (117)	
Missing	75.0 (3096)	93.4 (1463)	63.7 (1633)	
BMI (kg/m^2^)*				
Mean, SD	25.5 (3.9)	25.1 (4.6)	25.6 (3.8)	0.24
Missing, % (*N*)	76.5 (3160)	93.3 (1462)	66.3 (1698)	
Tumor level (cm)				
Mean (SD)	8.2 (1.8)	8.5 (1.7)	8.1 (1.8)	< 0.001
Cancer stage, % (*N*)				
I	31.7 (1309)	31.9 (500)	31.6 (809)	0.37
II	32.0 (1322)	33.1 (518)	31.4 (804)	
III	36.3 (1499)	35.0 (549)	37.1 (950)	
No. of positive lymph nodes, % (*N*)				
0	62.9 (1890)	64.9 (699)	61.7 (1191)	0.22
1–3	23.7 (714)	22.4 (241)	24.5 (473)	
≥ 4	13.4 (403)	12.7 (137)	13.8 (266)	
Missing	27.2 (1123)	31.3 (490)	24.7 (633)	
Neoadjuvant therapy, % (*N*)				
None	28.4 (1169)	37.1 (577)	23.2 (592)	< 0.001
Radiotherapy	67.5 (2775)	62.4 (971)	70.7 (1804)	
Radio plus chemotherapy	4.0 (165)	0.6 (9)	6.1 (156)	
Missing	0.5 (21)	0.6 (10)	0.4 (11)	
Intraoperative bleeding (mL)				
Median, (Q1– Q3)	600 (300; 1000)	500 (300; 900)	600 (350; 1000)	< 0.001
Missing, % (*N*)	21.7 (898)	34.5 (541)	13.9 (357)	
Symptomatic anastomotic leakage, % (*N*)				
No	88.5 (3656)	85.3 (1337)	90.5 (2319)	< 0.001
Yes	11.5 (474)	14.7 (230)	9.5 (244)	
Length of in hospital stay (days)				
Median, (Q1–Q3)	10 (8; 15)	9 (7; 14)	11 (8; 16)	< 0.001
Missing, % (*N*)	0.2 (10)	0.3 (5)	0.2 (5)	
30-day mortality, % (*N*)	1.4 (59)	2.3 (36)	0.9 (23)	< 0.001
90-day mortality, % (*N*)	2.5 (102)	3.6 (57)	1.8 (45)	< 0.001
Calendar year of surgery, % (*N*)				
1995–2000	31.1 (1283)	41.2 (646)	24.9 (637)	< 0.001
2001–2005	35.7 (1474)	46.3 (726)	29.2 (748)	
2006–2010	33.2 (1373)	12.4 (195)	46.0 (1178)	

*LAR*, low anterior resection; *SD*, standard deviation; *Q1*, quartile 1; *Q3*, quartile 3; *ASA*, American Society of Anesthesiologists; *BMI*, body mass index. *BMI and ASA are recorded in the SCRCR from 2007 onwards

*Number of lymph nodes was recorded in the SCRCR by some health care regions from 1995 and by all health care regions (*n* = 6) from 2003 onwards

Only a small number of patients, 0.5% (21/4130), had missing covariate data and all associations with oncological outcomes presented are therefore based on complete case data.

### Mortality and long-term oncological outcomes

**Fig. 2:**
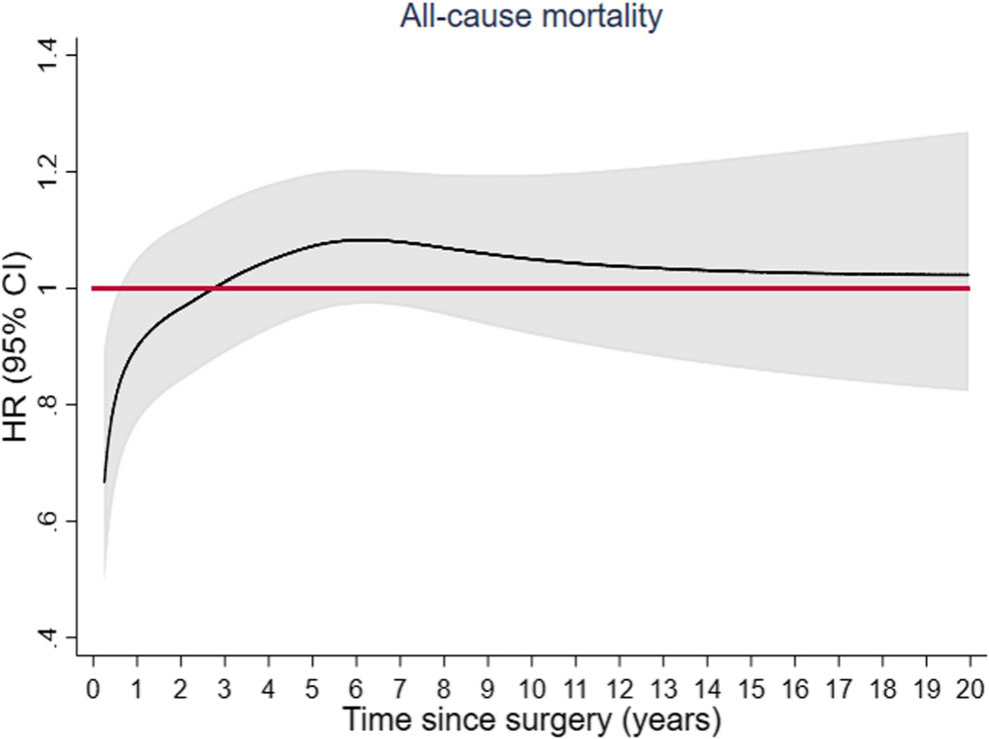
Time-dependent hazard ratios for all-cause mortality associated with defunctioning stoma at index surgery in patients operated with low anterior resection for rectal cancer. Time-dependent HRs are adjusted for age, sex, cancer stage, tumor level, neoadjuvant therapy, and year of surgery (model 2). CI, confidence interval; HR, hazard ratio

**Fig. 3:**
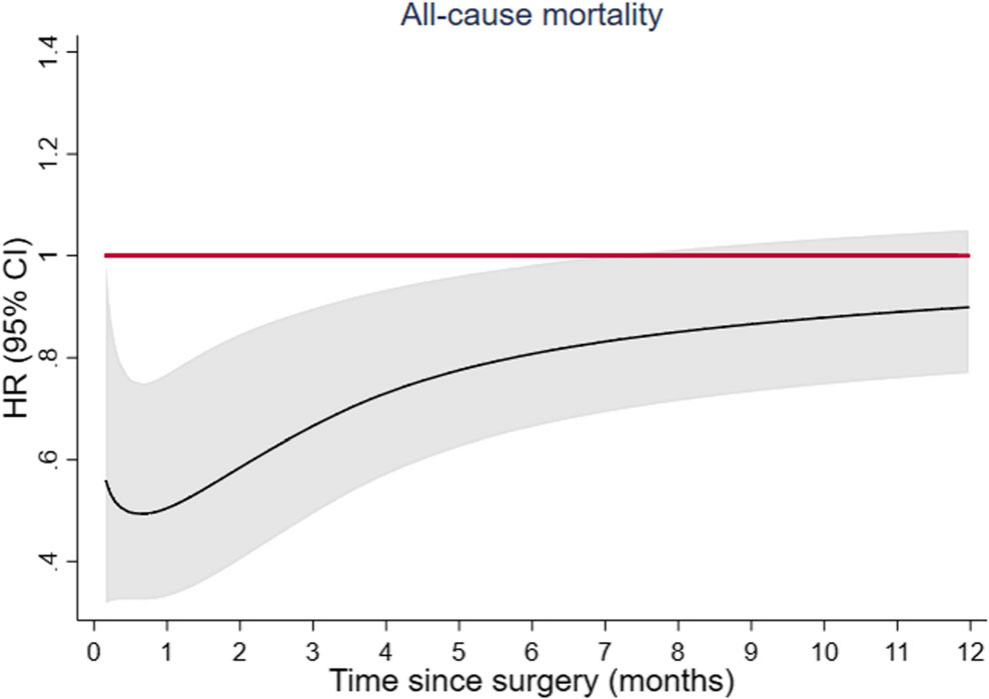
Time-dependent hazard ratios for all-cause mortality associated with defunctioning stoma at index surgery in the first year following surgery in patients operated with low anterior resection for rectal cancer. Time-dependent HRs are adjusted for age, sex, cancer stage, tumor level, neoadjuvant therapy, and year of surgery (model 2). CI, confidence interval; HR, hazard ratio

**Fig. 4:**
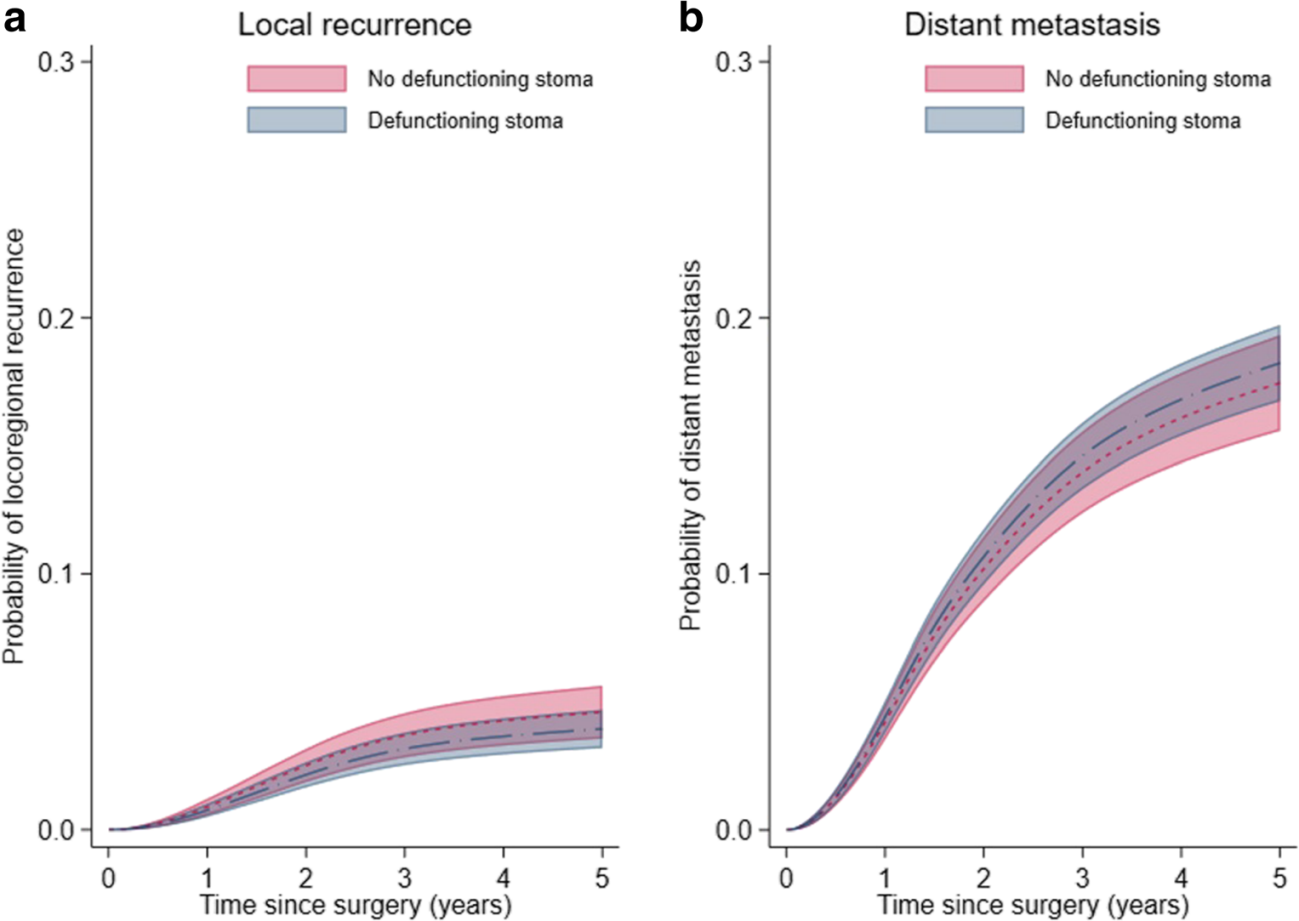
Cumulative incidence of local recurrence and distant metastatic disease in patients operated with low anterior resection for rectal cancer with and without defunctioning stoma at index surgery. Cumulative incidence curves with 95% confidence intervals for local recurrence and distant metastasis in patients with and without a defunctioning stoma were estimated from flexible parametric models assuming proportional hazards with death as competing event

Overall, 2169 patients died during a median follow-up of 8.3 (interquartile range 4.7 to 12.4) years. Tests for proportional hazards revealed that the association between defunctioning stoma and all-cause mortality was not constant over time. In analyses adjusting for year of surgery, patients with a defunctioning stoma had a lower mortality rate early after surgery than those without (HR at 6 months 0.79; 95% CI: 0.67 to 0.92, and at 1 year 0.81; 95% CI: 0.68–0.96), while no long-term difference in mortality was found (Tables 2 and 3; Figs. 2 and 3). Estimates were not very different in multivariable adjusted analyses except for the association with mortality only being statistically significant up to 6 months (HR at 6 months = 0.82; 95% CI 0.67 to 0.99 and at 1 year = 0.88 (0.75; 1.03)). After 5 years, 4.2% (173/4130) of the patients had a local recurrence registered and 17.9% (741/4130) had metastatic disease. No evidence of non-proportional hazards was found for these study endpoints. The cumulative incidence curves for local recurrence and distant metastatic disease by defunctioning stoma are presented in Fig. 4. The 5-year cumulative incidence for local recurrence in patients with and without a defunctioning stoma was 3.9% (95% CI: 3.2 to 4.7%) and 4.6% (95% CI: 3.6 to 5.6%), respectively. Corresponding estimates for distant metastatic disease were 18.3% (95% CI: 16.7 to 19.7%) and 17.5% (95% CI: 15.6 to 19.3%), respectively. Multivariable analyses yielded similar results, with no evidence of a risk difference in local recurrence (HR = 1.00; 95% CI: 0.72 to 1.39) and distant metastatic disease (HR = 1.03; 95% CI: 0.87 to 1.21) when comparing patients with and without a defunctioning stoma (Table 4).

**Table 2:** Hazard ratios for all-cause mortality associated with planned defunctioning stoma at different time points following surgery in patients operated with low anterior resection for rectal cancer

		HR (95% CI)					
	*N* all/deaths	At 6 months	At 1 year	At 2 years	At 5 years	At 10 years	At 15 years
Model 1 defunctioning stoma							
No	1567/940	REF	REF	REF	REF	REF	REF
Yes	2563/1229	0.75 (0.61; 0.92)	0.81 (0.68; 0.96)	0.90 (0.78; 1.05)	1.07 (0.94; 1.21)	1.07 (0.94; 1.22)	1.01 (0.81; 1.28)
Model 2 defunctioning stoma							
No	1557/933	REF	REF	REF	REF	REF	REF
Yes	2552/1221	0.82 (0.67; 0.99)	0.88 (0.75; 1.03)	0.97 (0.84; 1.11)	1.06 (0.95; 1.18)	1.05 (0.93; 1.20)	1.05 (0.89; 1.25)

HRs were estimated from flexible parametric models with time since surgery as underlying time scale, not assuming proportional hazards (allowing HRs to vary over time)

Model 1: adjusted for year of surgery

Model 2: adjusted for age, sex, cancer stage, tumor level, neoadjuvant therapy, and year of surgery

*HR*, hazard ratio; *CI*, confidence interval

**Table 3:** Multivariable adjusted hazard ratios for all-cause mortality associated with defunctioning stoma within the first year of follow-up in patients operated with low anterior resection for rectal cancer

	HR (95% CI)					
	At 1 month	At 2 months	At 3 months	At 4 months	At 5 months	At 6 months
Model 2 defunctioning stoma						
No	REF	REF	REF	REF	REF	REF
Yes	0.51 (0.32; 0.81)	0.60 (0.40; 0.91)	0.69 (0.51; 0.95)	0.76 (0.59; 0.97)	0.79 (0.64; 0.98)	0.82 (0.67; 0.99)
	At 7 months	At 8 months	At 9 months	At 10 months	At 11 months	At 12 months
Model 2 defunctioning stoma						
No	REF	REF	REF	REF	REF	REF
Yes	0.83 (0.70; 1.00)	0.85 (0.71; 1.01)	0.86 (0.73; 1.01)	0.87 (0.74; 1.02)	0.87 (0.74; 1.02)	0.88 (0.75; 1.03)

HRs were estimated from flexible parametric models with time since surgery as underlying time scale, not assuming proportional hazards (allowing HRs to vary over time)

Model 2: adjusted for age, sex, cancer stage, tumor level, neoadjuvant therapy, and year of surgery

*HR*, hazard ratio; *CI*, confidence interval

**Table 4:** Hazard ratios for local recurrence and distant metastasis associated with planned defunctioning stoma in patients operated with low anterior resection for rectal cancer

	Local recurrence		Distant metastasis	
	*N* all/events	HR (95% CI)	*N* all/events	HR (95% CI)
Model 1 defunctioning stoma				
No	1567/72	REF (1.00)	1567/274	REF (1.00)
Yes	2563/101	1.02 (0.74; 1.40)	2563/467	1.06 (0.90; 1.25)
Model 2 defunctioning stoma				
No	1557/72	REF (1.00)	1557/273	REF (1.00)
Yes	2552/100	1.00 (0.72; 1.39)	2552/466	1.03 (0.87; 1.21)

HRs were estimated from flexible parametric models with time since surgery as underlying time scale, assuming proportional hazards and accounting for the competing risk of death

Model 1: adjusted for year of surgery

Model 2: adjusted for age, sex, cancer stage, tumor level, neoadjuvant therapy, and year of surgery

*HR*, hazard ratio; *CI*, confidence interval

HRs for all study endpoints, i.e., all-cause mortality, local recurrence, and distant metastatic disease, were not notably different after additional adjustment for BMI and ASA except for confidence intervals being wider given the smaller number of patients and events included in this analysis (Tables 5 and 6).

**Table 5:** Hazard ratios for all-cause mortality associated with planned defunctioning stoma at different time points in patients operated with low anterior resection for cancer. Sensitivity analysis

		HR (95% CI)					
	*N* all/events	At 6 months	At 1 year	At 2 years	At 5 years	At 10 years	At 15 years
Model 3 defunctioning stoma							
No	100/42	REF	REF	REF	REF	REF	REF
Yes	851/230	0.51 (0.27; 0.97)	0.63 (0.38; 1.07)	0.80 (0.46; 1.41)	0.82 (0.54; 1.25)	0.72 (0.26; 2.00)	0.72 (0.19; 2.69)

HRs were estimated from flexible parametric models with time since surgery as underlying time scale, not assuming proportional hazards (allowing HRs to vary over time)

Model 3: adjusted for age, sex, BMI, ASA, cancer stage, tumor level, neoadjuvant therapy, and year of surgery in a subcohort of patients operated between 2007 and 2010

*HR*, hazard ratio; *CI*, confidence interval

**Table 6:** Hazard ratios for local recurrence and distant metastasis associated with planned defunctioning stoma in patients operated with low anterior resection for rectal cancer. Sensitivity analysis

	Local recurrence		Distant metastasis	
	*N* all/events	HR (95% CI)	*N* all/events	HR (95% CI)
Model 3 defunctioning stoma				
No	100/4	REF (1.00)	100/24	REF (1.00)
Yes	851/21	0.79 (0.23; 2.71)	851/148	0.71 (0.44; 1.14)

HRs were estimated from flexible parametric models with time since surgery as underlying time scale, not assuming proportional hazards (allowing HRs to vary over time)

Model 3: adjusted for age, sex, BMI, ASA, cancer stage, tumor level, neoadjuvant therapy, and year of surgery in a subcohort of patients operated between 2007 and 2010

*HR*, hazard ratio; *CI*, confidence interval

## Discussion

The present study demonstrates that short-term all-cause mortality was lower in patients undergoing LAR for cancer with a defunctioning stoma at index surgery, compared with that in those without an initial defunctioning stoma. More specifically, patients with a defunctioning stoma at index surgery had a lower mortality rate up to 6 months after surgery. No differences in long-term overall mortality, nor oncological outcome with regard to 5-year local recurrence or metastatic disease, were found between patients with and without a defunctioning stoma at index surgery.

### Strengths and limitations

This is a large study addressing long-term oncological outcomes in rectal cancer patients undergoing LAR in relation to the presence of a defunctioning stoma. The present study has several strengths. First, the nationwide register–based design with prospective data collection and complete follow-up in a large population-based sample minimizes the risk of selection and information biases. However, despite the high degree of coverage of the SCRCR, some shortcomings of the register have been reported [28, 32]. For instance, anastomotic level is not registered in the SCRCR and we therefore used the tumor height as a proxy for LAR. This strategy of excluding patients with a tumor level of more than 10 cm (*n* = 5025) may not have resulted in the exclusion of all patients with an anastomosis above the pelvic floor, perhaps more likely in women, but we deem that the vast majority of patients included in the present study in fact underwent LAR*.* A limited number of patients (*n* = 84) with a registered tumor height at 1–3 cm above the anal verge were arbitrarily excluded due to the potential risk of misclassification regarding surgical procedure (LAR registered instead of APE or possibly Hartmann’s procedure). Furthermore, SCRCR validation data for long-term oncological outcomes are limited. We acknowledge the risk of underreporting of local recurrences to some extent. However, any underreporting is unlikely to be related to whether a defunctioning stoma at index surgery was placed or not, and as such has probably resulted in an underestimation of associations observed as this weakens the validity of the negative finding in this study, due to random rather than systematic misclassification. Lastly, we adjusted our analyses for a comprehensive set of observed confounders, but as this is an observational study, we cannot rule out residual confounding.

### Comparison with previous studies

Evidence from randomized clinical trials shows that a defunctioning stoma reduces the rate of symptomatic anastomotic leakage in rectal patients undergoing LAR [10, 11, 33]. Consistent with this evidence, we found a lower anastomotic leakage rate in patients with a defunctioning stoma compared to those without (9.5% and 14.7%, respectively). This crude estimate corresponds to a relative risk reduction of 35%, which, although substantial, is lower than the reduction seen in randomized trials assessing defunctioning stomas in LAR [10, 11, 33]. Also, the crude estimate reported here is not directly comparable to estimates observed in randomized trials, which, by design, control for confounding by measured and unmeasured factors. Moreover, we acknowledge that there may be some underreporting of anastomotic leakage in the SCRCR, as in contrast to prospective randomized controlled trials. However, the use of defunctioning stomas in this study cohort was at the discretion of the surgical team, leading to a considerable case-mix, for instance a larger proportion of neoadjuvant therapy in the stoma group, which explains the relatively lesser degree of leakage reduction [10, 11].

As symptomatic anastomotic leakage is associated with severe adverse events such as peritonitis and sepsis, a short-term survival benefit in patients receiving a defunctioning stoma at index surgery is considered plausible. A defunctioning stoma also mitigates the clinical consequences when a symptomatic leakage occurs, and could thereby also reduce mortality in this particular group of patients. In our study, 30-day and 90-day mortality rates were lower in patients with a defunctioning stoma at index surgery compared to those without, a finding that is in agreement with meta-analysis results of observational data showing a consistent reduction in postoperative mortality in patients receiving a defunctioning stoma at index surgery [5, 13, 34].

Previous observational studies and randomized clinical trials could not address differences in mortality by time since surgery and were unable to investigate differences in mortality beyond the initial postoperative time window. The present study therefore extends the available evidence base by showing that the overall reduction in all-cause mortality in patients with a defunctioning stoma persists up to 6 months after surgery, but not thereafter.

Data concerning the association with disease recurrence are limited. A study by Smith et al., which used a similar register-based design, found no difference in 5-year disease recurrence and overall survival in patients with and without a defunctioning stoma in stratified analyses. The aforementioned study had a sample size of one-fourth of our study, but its results are consistent with ours showing no association with long-term outcome. The evidence base regarding the association of anastomotic leakage with long-term oncological outcome is larger but conflicting, with studies showing either no difference in risk or an increased risk of specific oncological outcomes, mostly local recurrence and to a smaller extent cancer-specific mortality [22–25, 35]. These discrepant results have been attributed to various factors, including the variability in diagnosis and management of leakages as well as heterogeneity in patient populations analyzed with the presence of residual disease being the strongest determinant of local recurrence risk [23, 36]. Findings observed for defunctioning stoma in our study appear to be consistent with results from studies in comparable rectal cancer patient populations with little evidence of residual disease, showing no difference in long-term oncological outcome with anastomotic leakage occurrence [22, 23].

### Implications

Results of the present study demonstrate that, despite a short-term mortality reduction, long-term all-cause mortality and disease recurrence are not affected by a defunctioning stoma in rectal cancer patients undergoing LAR. Overall, these findings support the use of a defunctioning stoma as standard of care in LAR. Nevertheless, a defunctioning stoma is associated with numerous adverse events which, although different in nature, are generally less severe than those associated with a symptomatic anastomotic leakage [15–21]. It therefore seems relevant to attempt to individualize the routine use of a defunctioning stoma or not, balancing the risks of the stoma against the benefit of reductions in symptomatic anastomotic leakage and short-term mortality, also from a cost-effectiveness perspective [37]. Future studies aiming at identifying patients in whom the preoperatively assessable risks do no outweigh the benefits using a defunctioning stoma are warranted, as well as research aimed at developing strategies to reduce the adverse events related to the defunctioning stoma itself.

## Conclusion

Our findings demonstrate that a defunctioning stoma is associated with a short-term reduction in all-cause mortality in patients undergoing low anterior resection for rectal cancer, without any difference in long-term mortality and oncological outcome, and should be considered standard of care.
